# Supplementary material for: Protective Effect of Cocoa Bean Shell against Intestinal Damage: An Example of Byproduct Valorization
Source: Antioxidants (Basel). 2021 Feb 12;10(2):280. doi: 10.3390/antiox10020280 (PMC7918452; doi:10.3390/antiox10020280)
Supplement: Supplementary file 1 [file antioxidants-10-00280-s001.pdf]

Table S1. Formulations of the control ice cream (IC) and ice cream fortified with CBS at 4% (CBS-IC).

**Ingredients**	**Composition (g/100g IC)**	
	**IC**	**CBS-IC**
Skim milk	51.5	59.3
Skim milk powder	2.5	2.5
Cream milk	23.5	11.5
Sucrose	12.0	12.0
Inverted sugar	2.5	2.5
Glucose syrup	2.5	2.5
Base Nevepann 50 mix	2.5	2.5
Cocoa powder	3.0	3.0
CBS flour	0.0	4.2

Different ingredients present in plain ice cream (IC) preparation and in ice cream fortified with 4% CBS powder (CBS-IC) are expressed as g component/100 g of ice cream.

Table S2. Chemical composition (mean ± standard deviation; n=3) of CBS, plain ice cream (IC), and fortified ice cream with 4% CBS powder (CBS-IC).

Components	CBS	IC	CBS-IC
Humidity (g/100g)	5.90 ± 0.04	58.02 ± 0.01	61.40 ± 0.03
Protein (g/100g dw)	20.90 ± 0.05	3.94 ± 0.07	5.20 ± 0.09
Total fat (g/100g dw)	2.30 ± 0.14	8.00 ± 0.14	4.25 ± 0.08
Carbohydrates (g/100g dw)	8.70 ± 0.09	28.33 ± 0.54	24.78 ± 0.32
Ash (g/100g dw)	7.95 ± 0.33	0.88 ± 0.21	1.37 ± 0.40
Total dietary fibre (g/100g dw)	54.30 ± 2.11	0.83 ± 0.10	3.00 ± 0.11
Soluble dietary fibre (g/100g dw)	12.80 ± 0.28	n.d.	n.d.
Insoluble dietary fibre (g/100g dw)	42.30 ± 0.34	n.d.	n.d.

n.d.; not determined

Table S3. Cell viability evaluation in different percentages of CBS extracts

LDH (% cell release)				
	**5% sample extract**		**10% sample extract**	
	**-**	**+ Oxy-mix**	**-**	**+ Oxy-mix**
**Control**	7.4 ± 0.1	9.4 ± 0.5	7.4 ± 0.1	9.4 ± 0.5
**IC**	6.9 ± 1.1	7.7 ± 3.3	16.0 ± 1.2**	16.4 ± 0.1**
**CBS-IC**	9.5 ± 1.2	8.7 ±2.6	15.3 ± 0.4**	15.4 ± 0.4**
**CBS**	8.8 ± 1.6	8.7 ± 3.2	14.9 ± 0.5**	15.8 ± 0.5**
	**30% sample extract**		**50% sample extract**	
		**+ Oxy-mix**		**+ Oxy-mix**
**Control**	7.4 ± 0.1	9.4 ± 0.5	7.4 ± 0.1	9.4 ± 0.5
**IC**	51.1 ± 0.4***	47.1 ± 7.7***	71.9 ± 0.7***	62.1 ± 3.2***
**CBS-IC**	33.0 ± 5.1***	32.0 ± 0.2***	66.0 ± 0.1***	83.4 ± 2.9***
**CBS**	43.3 ± 1.0***	33.5 ± 2.3***	74.9 ± 1.7***	72.8 ± 7.7***

LDH release was evaluated in the culture media of differentiated CaCo-2 cells pre-treated or not with different concentration of ice cream (IC), ice cream fortified with CBS at 4% (CBS-IC) or CBS and incubated with 60 µM Oxy-mix for 24 h. Control: untreated cells.
LDH was calculated as percentage referred to 100% cell enzyme released into the medium following the addition of 0.5% Triton X-100 to cultured cells grown at the same density of other samples. Data are reported as means ± SD of three independent experiments. Significantly different vs. controls: **$p<0.01$, ***$p<0.001$.

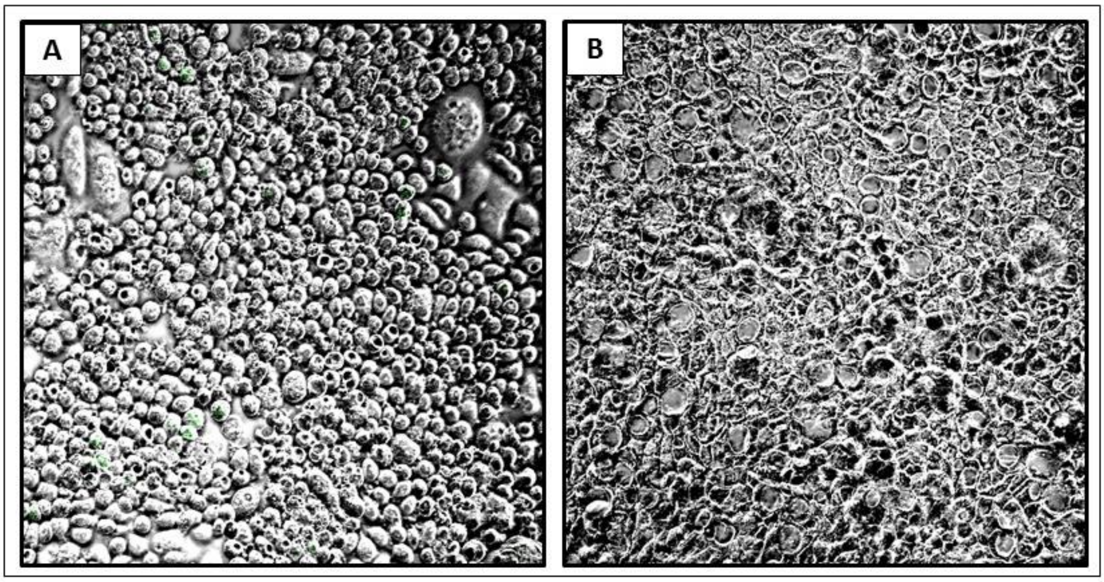

Figure S1. CaCo-2 Cell Imaging by Laser Scanning Confocal Microscopy.
CaCo-2 cells were visualized at two cell culture steps: A) at the confluence; B) at 18 post-confluence days. After reaching confluence CaCo-2 cells spontaneously began to differentiate and reach full differentiation after further18 days of culture (the so-called differentiated cells). The different morphology between undifferentiated- (A) and differentiated-CaCo-2 cells (B) was directly visualized on cell culture plates by laser scanning confocal microscopy (LSM 800 confocal laser microscope, Zeiss SpA, Oberkochen, Germany) equipped with a Zeiss inverted microscope, plane neofluar lens 20×/0.5. The instrument was set to 488 nm exciting laser band, with a 515 nm long pass emission filter. Images were elaborated using a Zeiss LSM 800 Image Examiner software.
